# Traumatic Brain Injuries: Comprehensive Management of Complex Clinical Scenarios

**DOI:** 10.1155/2023/9754321

**Published:** 2023-04-20

**Authors:** Mario Ganau, Antonio Belli, Timothy P. Lawrence, Chris Uff

**Affiliations:** ^1^Oxford University Hospitals, Oxford, UK; ^2^Nuffield Department of Clinical Neurosciences, University of Oxford, Oxford, UK; ^3^University of Birmingham, Birmingham, UK; ^4^Queen Mary University of London, London, UK

The number of traumatic brain injuries (TBI) is on the rise worldwide, representing 30–40% of injury-related deaths in many countries [1, 2]. TBI is effectively a public health epidemic; this justifies the 6-fold increase in the number of studies published over the last decade [3]. As such, the impact of TBI on the global burden of disease (GBD) is substantial, according to the investigators of the GBD study who annually quantify health loss from all types of diseases and injuries with the aim of improving health systems all over the world, and in the long term helps in reducing/eliminating disparities [1, 2, 4].

Due to groundbreaking mechanistic studies and randomized clinical trials (RCT) published in recent years (accounting for more than 380 new PubMed-indexed RCT on TBI since 2019), the approach to neurotrauma patients has remarkably evolved with an increase in the overall quality of acute care. One of the reasons for this improvement is certainly the care provided by a broad spectrum of multidisciplinary team (MDT) specialists, including prehospital and emergency department (ED) doctors and nurses, neuroradiologists, neurosurgeons, anesthetists, and intensive care physicians. Conceived and addressed to those professionals, this Special Issue has gathered insightful quantitative and qualitative studies on various aspects of the basic sciences, ethics and clinical practice of TBI, contributing to the existing body of literature, reinforcing data from recent trials and covering existing knowledge gaps. This editorial is meant to summarize the main findings from this collection and its overall achievements.

First of all, the greatest measure of this Special Issue's success can be measured by the outreach on authors from both high-income countries (HIC) and low- and middle-income countries (LMIC). In fact, the major contributors to this collection were authors from the United Kingdom and Turkey, heavily involved into academic research in the field of global neurotrauma, and authors from sub-Saharan African countries, such as Cameroon and Ethiopia, who shared their practical experience on the management of adult and pediatric patients with TBI in remote geographical locations. The number of contributions from LMIC, where the epidemiological distribution of TBI is the highest, represents indeed a particularly relevant factor because we know that mechanisms of injury, referral pathways, and access to tertiary centers are vastly different among continents, and the challenges faced for TBI management in LMIC are quite different from those of HIC [5]. Among the reasons for limited resources available in LMIC, and sources of concern for the global neurotrauma community, is the paucity of neurosurgical workforce requiring a remarkable task shifting and sharing practices [6]. This aspect has obviously an impact on hospitals preparedness, as recently shown in a collaborative study published by the NIHR Global Health Unit on Global Surgery [7]. Hence, the first-hand experience from LMIC contributors was obviously deemed extremely valuable and welcomed by our Editorial Board in the context of this Special Issue.

Above all, one aspect that was underscored in this collection reflects the organizational heterogeneity across centers dealing with high volumes of trauma referrals. This emerged quite well in the observational cross-sectional study from Bedry and Tadele [8], which provided data on the clinical profile and outcome of childhood TBI at a tertiary hospital in Southern Ethiopia where head injuries contribute to 7.4% of pediatric visits in the local EDs. Of note, their clinical series indicated that road traffic accidents (RTA) and falls represent the most common causes of TBI in LMIC, as recently confirmed by Dewan et al. who estimated that the proportion of TBIs resulting from RTA in Africa and Southeast Asia is up to 56% of the total cases of head injuries registered in those continents [9]. Besides the organizational challenges, both articles explored critical aspects allowing clinicians to prognosticate outcome: both concluded that prolonged hospital stay and poor outcome correlate with comorbid illness, loss of consciousness at presentation, increased ICP sign, severity of head injury, presence of seizures, hypotension, and hyperglycemia on presentation.

While early hyperglycemia mentioned in their list of risk factors is a known predictor of mortality and correlates with mechanisms of secondary injury, as previously shown by European studies conducted in the acute phase by Prisco et al. [10] and in the subacute phase by TRACK-TBI investigators [11], other serum, plasma, and cerebrospinal fluid (CSF) markers of inflammatory reaction have also emerged in recent times [12–15]. Some useful biomarkers were extensively discussed in this Special Issue: for instance, the narrative review from Erenler and Baydin [16] indicates that IL-33 has emerged, among multiple plasma biomarkers, as the one mostly implicated in cellular crosstalk and responsible for multiorgans impairment. IL-33 is in fact a powerful endogenous alarm signal (alarmin) meant to alert various types of immune cells to trauma, and the study from Erenler and Baydin allowed revisiting data from experimental preclinical models of TBI [17, 18] and case-control studies [19, 20] on TBI patients, concluding the great potential role of IL-33 as an early indicator of secondary injury.

Together with contributions exploring prognostic factors and the role of biomarkers, this Special Issue includes more studies attempting to answer the demand for new pharmacological treatments. This was the case in the article written by Nguembu et al. [21] who explored the implication of paroxysmal sympathetic hyperactivity triggered by TBI, which could be effectively tackled by innovative neuroprotective strategies based on well-known, conventional drugs such as beta-blockers. Their scoping review suggests that beta-blockers diminish the effect of circulating catecholamines and attenuate the resting metabolism rate, which is markedly increased in patients with severe acute brain injury [22–29]. As such, their conclusions were that propranolol and labetalol should have a greater role in the acute management of TBI [30, 31]. Speaking of pharmacological strategies, another long-term retrospective study from Acar et al. [32] investigated the use of tranexamic acid (TXA) in blunt and penetrating TBI in the context of poly-traumas. Their study design represented a pragmatic approach to the use of antifibrinolytic drugs in the treatment and prevention of major bleeding. Conducted between 2012 and 2020, the work from Acar et al. reached a conclusion about the safety of TXA in TBI (none of the 51 patients included had thrombotic complications nor died due to head injury), which is in keeping with the main findings from the CRASH 2 and the more recent CRASH 3 trials [33, 34]. Additionally, the article from Acar et al. offers a much needed confirmation in a civilian ED environment of the findings from the battlefield reported by Dixon et al. [35] and the results obtained within the constraints of the abovementioned RCT.

In terms of surgical strategies for TBI, with a specific focus for those developed in LMIC, Kanmounye [36] focused his attention to the rise of inflow cisternostomy, a more modern alternative to the 1940 ideas of outflow cisternostomy in the form of either ventriculocisternostomy and cystocisternostomy [37, 38]. Starting from the first description of such a surgical technique for the management of severe TBI, which dates back to the 2012 article by Dr. Cherian from Nepal [39], Kanmounye, who is also the founding President of the Association of Future African Neurosurgeons, provided an historical vignette of the evolution of such a technique in limited resource settings and offered a detailed argumentation for its rationale, limitations, and future challenges. That article highlighted that the use of cisternostomy in the surgical management of severe TBI certainly represents a revolutionary step, and we definitely agreed with the statement that the disruptive theory of CSF shift edema behind its conception has already contributed lessons to the entire neurotrauma community [40].

As mentioned in this Special Issue's call for papers new imaging tools, validated surgical strategies and optimized protocols for clinical follow-up, the early resuscitation, and management of difficult cases still represent a clinical, surgical, and ethical challenge most of the time. This is possibly the reason why one of the articles, which received most attention, earning double digits citations (the highest so far for this collection), was the contribution from Hasan et al. [41] revolving on public engagement as a tool to validate research questions and protocols in the management of TBI patients. Their qualitative study, consisting in a survey submitted to severe TBI survivors and their next of kin, helped identifying ways to direct future research into more accurate prognostic models and therapeutic options in the acute and subacute phases of TBI management. Furthermore, they explored the complex ethical aspects of dealing with sensitive issues revolving around TBI and the challenges faced in the aftermath of major trauma, not only by patients but also by clinicians and scientists.

Given the quality of the submissions received, it is no surprise that at time of writing this editorial, our Special Issue has gathered a cumulative number of 15,789 visualizations and a total of 8,111 downloads. Those results not only testify the valuable insights from both a scientific and teaching perspective offered by the authors who joined this endeavor but also the fulfillment of our initial goal of reaching out to and hopefully go beyond the vast scientific community of Emergency Medicine International.

*Mario Ganau* *Antonio Belli* *Timothy P. Lawrence* *Chris Uff*
